# Chemotherapy-induced gut microbiota dysbiosis exacerbates cancer-related fatigue in breast cancer patients via neuroimmune-endocrine indicators

**DOI:** 10.3389/fonc.2026.1710457

**Published:** 2026-01-26

**Authors:** Fengxia Lai, Yang Yuan, Haiyan Dong, Daoxia Guo, Zhongfang Yang, Li Tian

**Affiliations:** 1 The First Affiliated Hospital of Soochow University, Suzhou, China; 2 School of Nursing, Medical College of Soochow University, Suzhou, China; 3 925th Hospital of People's Liberation Army (PLA) Joint Logistics Support Force, Guiyang, China

**Keywords:** adjuvant chemotherapy, breast cancer, cancer-related fatigue, differential abundance analysis, gut microbiota, neuroimmune-endocrine indicators

## Abstract

**Background:**

Breast cancer patients frequently experience debilitating cancer-related fatigue (CRF) during chemotherapy. Emerging evidence implicates the gut microbiota (GM) and the gut-brain axis in CRF pathogenesis, yet whether pre-chemotherapy GM profiles can predict CRF remains unclear.

**Methods:**

This prospective cohort study enrolled 100 breast cancer patients initiating chemotherapy. GM profiling and fatigue assessment (Visual Analogue Fatigue Scale, Cancer Fatigue Scale) were performed at baseline and the third chemotherapy cycle. Serum levels of neuroimmune-endocrine markers were also measured. Multivariate logistic regression was used to build a predictive model for moderate-to-severe CRF.

**Results:**

Patients experiencing moderate-to-severe CRF at the third chemotherapy cycle demonstrated higher baseline *Bacteroidetes/Firmicutes* ratios, increased *Proteobacteria/Enterobacteriales* levels, and reduced abundance of short-chain fatty acid-producing bacteria. The predictive model incorporating baseline GM signatures and clinical covariates achieved an AUC of 0.82, demonstrating good predictive accuracy for moderate-to-severe CRF. Decreased levels of *Firmicutes/Blautia* in the gut mucosal microenvironment, along with reduced serum brain-derived neurotrophic factor (BDNF), were associated with increased CRF.

**Conclusion:**

Baseline GM characteristics predict the risk and severity of chemotherapy-induced CRF, potentially through modulation of neuroimmune-endocrine pathways via gut-brain axis. These findings underscore the potential role of GM as a predictive biomarker and a therapeutic target for chemotherapy-induced CRF.

## 1 Introduction

Breast cancer (BC) is the most prevalent malignancy in women globally and the fourth leading cause of cancer-related mortality (1). It frequently requires adjuvant chemotherapy, which is associated with debilitating side effects. Among these, cancer-related fatigue (CRF) emerges as one of the most common and distressing complications, substantially impairing quality of life (QoL) (2). The National Comprehensive Cancer Network (NCCN) defined CRF as a persistent and distressing sense of physical, emotional, and/or cognitive tiredness that is disproportionate to recent activity and interferes with usual functioning (3). CRF affects more than 90% of patients receiving chemotherapy (4), with BC patients experiencing higher incidence and severity due to intensive multimodal treatment regimens (5). Notably, up to 30% of BC survivors endure severe fatigue for years after treatment, limiting physical capacity, delaying occupational reintegration, and affecting psychosocial well-being (6). Moreover, CRF severity independently predicts poorer QoL and reduced survival across cancer types (7). Despite its clinical significance, the pathophysiology of CRF remains poorly understood, and effective evidence-based interventions are lacking. This knowledge gap underscores the urgent need to elucidate the molecular mechanisms and identify potential therapeutic targets.

Emerging evidence underscores the pivotal role of GM homeostasis in systemic health, suggesting its potential involvement in CRF pathogenesis (8). The GM is a complex microbial ecosystem that regulates immune function, neuropsychological processes, and metabolic signaling between the gut and brain (9). Chemotherapy has been demonstrated to disrupt GM composition, leading to dysbiosis (10). Dysbiosis may compromise the intestinal barrier, allowing microbial products and neuroactive metabolites to enter systemic circulation (11). These changes can activate neural, immune, and endocrine pathways along the gut-brain axis, leading to the release of specific cytokines and active substances (e.g., brain-derived neurotrophic factor, nerve growth factor, γ-aminobutyric acid, interleukin-6, citrulline, lipopolysaccharide, low-density lipoprotein), contributing to neuroinflammation and central nervous system alterations (12). This cascade may ultimately manifest as fatigue and associated behavioral changes, such as reduced physical activity and social withdrawal. Therefore, identifying the predictive role of gut microbiota for the occurrence of CRF in breast cancer patients during chemotherapy is beneficial for the early prevention and intervention of CRF.

Cross-sectional studies have revealed differences in gut microbial diversity among patients experiencing varying severity of cancer-related fatigue (CRF) during chemotherapy (13). However, it remains unclear whether the pre-chemotherapy gut microbiota composition influences the development of CRF throughout treatment. Accumulating evidence suggests that gut microbiota-targeted strategies may help predict and prevent chemotherapy-induced behavioral side effects (14). Therefore, investigating the predictive role of baseline gut microbiota in CRF onset is warranted, with the aim of identifying microbial targets for interventions to mitigate both the incidence and severity of fatigue. Longitudinal studies indicate that CRF severity fluctuates across chemotherapy cycles, often peaking during mid-treatment (15), underscoring the need for early identification and intervention—particularly around the third chemotherapy cycle, when CRF burden is typically most pronounced and clinically relevant. Nevertheless, most existing studies are limited by cross-sectional designs, insufficient exploration of underlying mechanisms, and a lack of data specific to breast cancer populations undergoing chemotherapy.

To address these gaps, this study introduces a prospective cohort design with two key methodological strengths (1): collection of baseline GM profiles prior to chemotherapy to evaluate their genuine predictive value for CRF severity at the anticipated peak (third cycle), and (2) inclusion of a longitudinal pilot sub-study with paired fecal and blood samples to explore potential neuroimmune-endocrine pathways. This approach moves beyond cross-sectional association to offer insights into predictive biomarkers and potential gut-brain axis mechanisms in a clinically relevant BC population undergoing chemotherapy.

## 2 Methods

The study was approved by the Ethics Committee of Soochow University, with the ethics approval number SUDA20201221H02.

### 2.1 Participants and study design

Between January and October 2021, patients with BC who met predefined inclusion criteria were recruited from two tertiary grade-A hospitals in Suzhou, China. Inclusion criteria were as follows: a confirmed pathological diagnosis of BC, initiation of adjuvant chemotherapy (four circles) for the first time, age ≥ 18 years, stable dietary habits during the preceding six months, and providing signed informed consent. Exclusion criteria included a history of mental disorders, gastrointestinal diseases, hematological or other endocrine system disorders, and use of antibiotics or probiotics within the past three months. A total of 100 patients with BC were enrolled in this prospective study.

### 2.2 Outcome measures

At baseline (prior to the first chemotherapy cycle), participants underwent comprehensive data collection including demographic information, fatigue severity assessments using the Cancer Fatigue Scale (CFS) and Visual Analogue Fatigue Scale (VAFS; 0–10 scale), and fecal sample collection for GM profiling. According to previous research findings, patients with BC undergoing four cycles of chemotherapy typically experience peak CRF during the third chemotherapy cycle (16). Follow-up assessments were conducted during the third chemotherapy cycle. A VAFS score ≥ 4 was considered indicative of moderate-to-severe CRF that significantly affects daily functioning and requires target interventions beyond routine health education. Therefore, the occurrence of moderate-to-severe CRF (VAFS ≥ 4) at the third chemotherapy cycle was used as the primary event to investigate the association between baseline GM composition and CRF severity in patients with BC.

#### 2.2.1 General information

According to the literature, factors influencing CRF in patients with cancer may include sociodemographic characteristics (e.g., gender, age, education level, employment status, marital status, social relationships, and income), cancer type and treatment regimen, and physical conditions (e.g., obesity, disability, menopause, pain, and sleep quality) (17–19).

To minimize the impact of confounding factors, a general information questionnaire was designed based on a comprehensive review of the literature, combined with clinical practice experience and expert consultation. Sociodemographic data included age, religious belief, marital status, education level, employment status, living situations, per capita monthly household income, and method of medical expense payment. Clinical data included body mass index (BMI), menstrual status, family history, comorbidities, pain, sleep quality, cancer stage, chemotherapy regimen, concurrent radiotherapy, and presence of metastasis.

#### 2.2.2 Cancer-related fatigue measures

##### 2.2.2.1 Cancer Fatigue Scale

Designed by Japanese scholar Okuyama in 2000 (20), the Cancer Fatigue Scale (CFS) is a widely used tool for assessing fatigue symptoms in patients with cancer. The scale comprises three dimensions: physical fatigue, emotional fatigue, and cognitive fatigue, including a total of 15 items. Each item is rated on a 5-point Likert scale (1 to 5), with total scores ranging from 0 to 60. Higher scores indicate more severe fatigue. The scale was translated into Chinese by Zhang (21). In validation studies, the Chinese version demonstrated good internal consistency, with Cronbach’s α coefficients for each dimension and the overall scale ranging from 0.63 to 0.86. Test-retest reliability coefficients ranged from 0.55 to 0.77, supporting its satisfactory reliability and validity in Chinese populations.

##### 2.2.2.2 Visual Analog Fatigue Scale

The Visual Analog Fatigue Scale (VAFS) is used to assess a patient’s level of fatigue using a ruler with 11 gradations, ranging from “I do not feel tired” to “I feel exhausted”. VAFS employs a numerical scale of 0-10, where 0 indicates no fatigue, 1–3 indicates mild fatigue, 4–6 indicates moderate fatigue, and 7–10 indicates severe fatigue. In this study, patients were categorized into the mild fatigue group (< 4 points) and the moderate-to-severe fatigue group (≥ 4 points).

#### 2.2.3 Fecal sample collection and analysis

##### 2.2.3.1 GM analysis

GM analysis was conducted using fecal samples. Fresh stool samples were collected in GUHE Flora Storage buffer (GUHE Laboratories, Hangzhou, China). Within 15 minutes after defecation, approximately a 1g of stool sample (about the size of a soybean) was collected using a sterile collection spoon and transferred into a sterile collection tube containing preservation solution, ensuring complete immersion of the sample. The tube was then tightly sealed, and samples were stored at −80 °C until analysis.

##### 2.2.3.2 16S rDNA amplicon pyrosequencing and bioinformatics analysis

Whole bacterial genomic DNA was extracted using the GHFDE100 DNA isolation kit (Hangzhou Guhe Information Technology Co., Ltd., Hangzhou, China). Nucleic acid quantification was performed using a NanoDrop microspectrophotometer (Thermo Scientific, 2000c) and a Qubit 2.0 fluorometer (Life Technologies, Q32866).

Specific primers with barcodes targeting the V4 region of the 16S rDNA gene were synthesized and used for PCR amplification (primers were diluted to 1 µM in nuclease-free water before use). After purification, DNA quantification was performed using the Qubit dsDNA HS Assay Kit (QIAGEN: 28706). Sequencing was conducted on a high-throughput platform (Illumina NovaSeq 6000).

Bioinformatics analysis was primarily performed using VSearch software (v2.4.4). Samples were distinguished based on barcode and primer sequences. At a 97% similarity threshold were clustered into operational taxonomic units (OTUs). Representative sequences were selected from each OTU using default parameters and taxonomically annotated using the SILVA128 database integrated in VSearch. Relative abundance and composition of OTUs were calculated at the phylum, class, order, family, and genus levels. OTUs representing less than 0.001% of the total sequences were excluded from analysis.

#### 2.2.4 Blood sample collection and analysis

##### 2.2.4.1 Neuroimmune-endocrine indicator measures

Blood samples were collected utilizing disodium ethylenediaminetetraacetic acid (EDTA) as an anticoagulant. Samples were centrifuged, and the resulting supernatant was extracted and stored at -80 °C in a cryogenic freezer until analysis. Following the manufacturer’s instructions, the serum levels of neuroimmune-endocrine indicator were assessed using the BDNF ELISA kit, NGF ELISA kit, OxLDL ELISA kit, GABA ELISA kit, Cit ELISA kit, and LPS ELISA kit, respectively. Experimental procedures were performed following the provided kit instructions (USCN, Wu Han, China), and standard curves were constructed. Subsequently, serum concentrations of BDNF, NGF, OxLDL, GABA, Cit, and LPS were determined by referencing these standard curves.

### 2.3 Statistical analyses

Data were analyzed using IBM SPSS Statistics version 25.0. Continuous variables were expressed as mean ± standard deviation (SD) and compared between groups using independent-sample t test. Categorical variables were reported as frequencies (percentages) and compared using the chi-square test or Fisher’s exact test, as appropriate.

GM sequencing data were processed using QIIME and R (v3.2.0). Differences in taxonomic composition from phylum to genus levels were analyzed using Kruskal-Wallis tests and illustrated with relative abundance bar plots. Alpha diversity indices (Chao1, Shannon, Simpson) were calculated in QIIME. Intergroup differences in alpha diversity were evaluated using Wilcoxon rank-sum tests and visualized via boxplots with corresponding t test. Intergroup differences in microbial community structures were assessed using principal coordinates analysis (PCoA) based on the Bray-Curtis dissimilarity metric, weighted UniFrac distance and unweighted UniFrac distance. Significant separation between groups was tested via ANOSIM and PERMANOVA test. LEfSe (Linear Discriminant Analysis Effect Size) was applied to identify differentially abundant taxa, integrating Kruskal-Wallis and Wilcoxon tests. Microbial functional predictions were conducted using PICRUSt (Phylogenetic Investigation of Communities by Reconstruction of Unobserved States) (22) and analyzed in STAMP.

To formally assess the predictive capacity of baseline gut microbiota for CRF severity, a multivariate logistic regression analysis was performed. Predictor variables were selected based on prior differential abundance analyses and differential species analysis. Clinically established covariates, such as age, body mass index, cancer stage, and chemotherapy regimen, were forced into the model to adjust for potential confounding. Model performance was evaluated using the area under the receiver operating characteristic curve (AUC-ROC), with sensitivity and specificity reported at the optimal cut-off point. The Hosmer-Lemeshow test was used to assess calibration.

To further validate our findings, a characteristic subgroup of 13 patients was selected from the 100 participants using stratified random sampling.

For correlations among CRF, neuroimmune-endocrine markers, and GM, CRF trajectories were categorized as “decreased/unchanged” or “increased” to balance group sizes. Δ values (third cycle – baseline) for neuroimmune-endocrine markers and bacterial taxa were computed. Between-group differences in Δ values were tested using the t-test or Mann-Whitney U test. Spearman’s rank correlation was used to assess associations among CRF, neuroimmune-endocrine markers, and GM at baseline and the third cycle. All correlations were adjusted using Benjamini-Hochberg correction. Statistical significance was set at *P <* 0.05. Detailed bioinformatics and statistical protocols are provided in the Supplementary Materials.

## 3 Results

### 3.1 Patient characteristics

All 100 patients with BC included in this study were female, with an average age of 50.95 ± 11.62 years. Based on the VAFS score at the third chemotherapy cycle, 42 patients were classified into the mild fatigue group (Y0), and 58 patients into the moderate-to-severe fatigue group (Y1). There were no significant differences in characteristics between the two groups (*P* > 0.05), indicating comparability (**Table 1**).

**Table 1:** Comparison of sociodemographic and disease-specific information between two groups of breast cancer patients.

Variable [*n* (%)]	Y0 (*n* = 42)	Y1 (*n* = 58)	*t/χ^2^*	*P*
Age, mean ± SD, yr	49.24 ± 11.37	52.19 ± 11.75	-1.257 ^a^	0.212
Religious belief			0.000 ^b^	1.000
Yes	3 (7.1)	3 (5.2)		
No	39 (92.9)	55 (94.8)		
Marital status			0.881 ^c^	1.000
Married/cohabitation	41 (97.6)	55 (94.8)		
Single/Divorced/Widowed	1 (2.4)	3 (5.2)		
Educational level			1.533 ^b^	0.465
Elementary school and below	13 (31.0)	20 (34.5)		
Middle school	21 (50.0)	32 (55.2)		
Junior college and above	8 (19.0)	6 (10.3)		
Employment status			1.064 ^b^	0.587
Employed	16 (38.1)	17 (29.3)		
Retired	11 (26.2)	15 (25.9)		
Unemployed	15 (35.7)	26 (44.8)		
Place of residence			0.001 ^b^	0.971
Urban	31 (73.8)	43 (74.1)		
Rural	11 (26.2)	15 (25.9)		
Lifestyle			0.000 ^b^	1.000
Living alone	2 (4.8)	3 (5.2)		
Living with family	40 (95.2)	55 (94.8)		
Per capita monthly household income			1.603 ^b^	0.449
<3000	11 (26.2)	14 (24.2)		
3000~6000	11 (26.2)	22 (37.9)		
>6000	20 (47.6)	22 (37.9)		
BMI, mean ± SD, kg/m^2^	23.83 ± 3.86	23.63 ± 3.71	0.265 ^a^	0.792
Menstrual condition			4.150 ^c^	0.241
Regular menstrual cycle	18 (42.9)	22 (37.9)		
Irregular menstrual cycle	5 (11.9)	6 (10.4)		
Menopause/Hysterectomy	19 (45.2)	30 (51.7)		
Family history			0.000 ^b^	0.986
Yes	2 (4.8)	4 (6.9)		
No	40 (95.2)	54 (93.1)		
Cancer metastasis			2.391 ^b^	0.122
Yes	17 (40.5)	15 (25.9)		
No	25 (59.5)	43 (74.1)		
Cancer Staging			0.191 ^c^	1.000
I	4 (9.5)	5 (8.6)		
II	34 (81.0)	48 (82.8)		
III	4 (9.5)	5 (8.6)		
Chemotherapy regimen			0.532 ^c^	0.596
TC	23 (34.3)	10 (30.3)		
EC	27 (40.3)	12 (36.4)		
AC	17 (25.4)	10 (30.3)		
Combined radiotherapy			0.000 ^b^	1.000
Yes	4 (9.5)	6 (10.3)		
No	38 (90.5)	52 (89.7)		
Other diseases			5.095 ^c^	0.083
Hypertension	5 (11.9)	17 (29.3)		
Diabetes	1 (2.4)	3 (5.2)		
None	36 (85.7)	38 (65.5)		
Pain score			3.151 ^c^	0.201
Painless	27 (64.3)	27 (46.6)		
Mild pain	14 (33.3)	29 (50.0)		
Moderate pain	1 (2.4)	2 (3.4)		
Sleep quality			1.492 ^c^	0.522
Good	8 (19.0)	17 (29.3)		
Average	30 (71.4)	37 (63.8)		
Poor	4 (9.6)	4 (6.9)		

Y1, VAFS≥4 points in the third cycle of chemotherapy; Y0, VAFS<4 points in the third cycle of chemotherapy; TC, docetaxel and cyclophosphamide; EC, paclitaxel and carboplatin; AC, doxorubicin plus cyclophosphamide; ^a^t-test; ^b^chi-square test; ^c^Fisher’s exact test.

### 3.2 Differences between mild fatigue group and moderate-to-severe fatigue group

#### 3.2.1 Cancer-related fatigue outcomes

At baseline, there were no significant differences between the Y0 and Y1 groups in physical fatigue, cognitive fatigue, total fatigue score (CFS score), and degree of fatigue (VAFS score) (*P >* 0.05). However, at the third chemotherapy cycle, significant differences were observed between the two groups in physical fatigue, emotional fatigue, total fatigue score, and degree of fatigue (*P* < 0.05), with all scores being higher in the Y1 group compared to the Y0 group (**Supplementary Table**, **Supplementary Table S1**).

#### 3.2.2 GM diversity and composition

**Figure 1:**
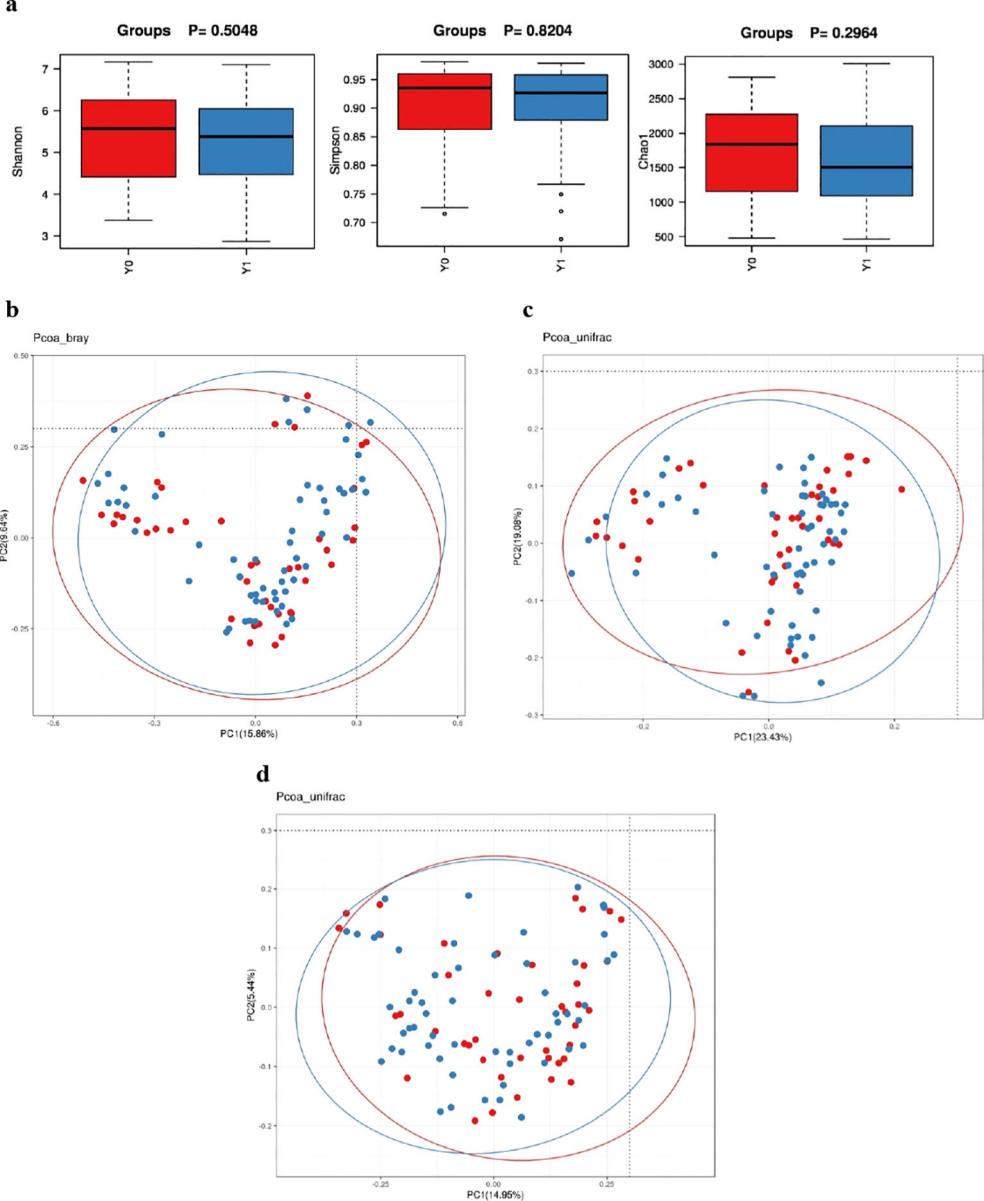
Alpha-diversity and Beta-diversity of gut microbiota in the Y1 group and Y0 group. **(a)** Shannon, Simpson and Chao1 indices; **(b)** Bray-Curtis distance; **(c)** weighted UniFrac distance; **(d)** unweighted UniFrac distance; Y1, VAFS24 points in the third cycle of chemotherapy; Y0, VAFS<4 points in the third cycle of chemotherapy.

**Figure 2:**
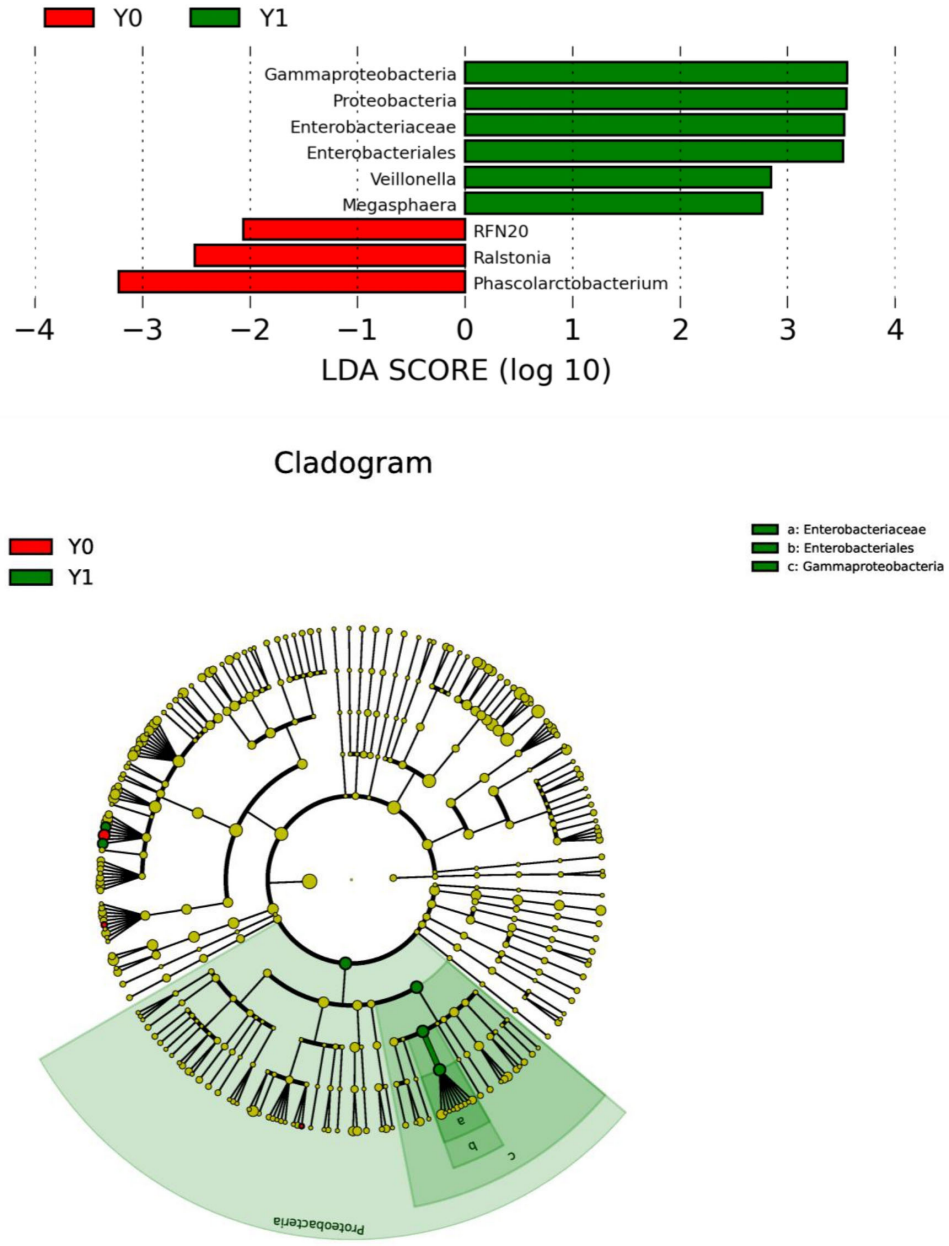
LEfSe analysis of the gut microbiota between the two patient groups. LEfSe, linear discriminant analysis effect size; LDA, linear discriminant analysis; Y1, VAFS24 points in the third cycle of chemotherapy; Y0, VAFS<4 points in the third cycle of chemotherapy.

Phylum-level analysis revealed *Bacteroidetes, Firmicutes, Proteobacteria, and Fusobacteria* as the predominant phyla in both groups, collectively accounting over 95% of the total GM composition (**Supplementary Figure**, **Supplementary Figure S1**). The *Bacteroidetes*/*Firmicutes* (B/F) ratio was significantly higher in the Y1 group than in the Y0 group (1.15 vs. 1.03, *P =* 0.043). The Y1 group also exhibited a higher abundance of *Proteobacteria* (8.03% vs 5.01%, *P* = 0.039). This was further reflected in increased relative abundances of *Gammaproteobacteria* (*P* = 0.015), *Enterobacteriales* (*P* = 0.026), and *Enterobacteriaceae* (*P* = 0.026). There was no significant difference in alpha-diversity between the Y0 and Y1 groups, according to the Shannon (*P =* 0.505), Simpson (*P =* 0.820), and Chao1 (*P =* 0.326) indices (**Figure 1**). Similarly, the beta-diversity weighted distance showed no significant difference between the Y0 and Y1 groups based on the ANOSIM (unweighted: *P =* 0.239, weighted: *P =* 0.492) and PERMANOVA (Bray-Curtis: *P =* 0.330, unweighted UniFrac: *P =* 0.841, weighted Unifrac: *P =* 0.551) analyses. Principal coordinate analysis (PCoA) results for the Y0 and Y1 groups are shown in **Figure 1**. LEfSe analysis showed that *Gammaproteobacteria, Proteobacteria, Enterobacteriaceae, Enterobacteriales, Veillonella*, and *Megasphaera* were more abundant in the Y1 group, whereas *RNF20, Rumiinococcaceae*, and *Phascolarctobacterium* were more abundant in the Y0 group (**Figure 2**).

#### 3.2.3 GM logistic regression model

The logistic regression model, incorporating both microbial and clinical predictors, demonstrated a significant ability to discriminate between patients who would develop mild versus moderate-to-severe CRF at T1. The final model achieved an AUC of 0.82 (95% CI: 0.73 - 0.90, *P* < 0.001), indicating good predictive accuracy (**Supplementary Figure**, **Supplementary Figure S2**). At the optimal probability cut - off of 0.49, the model exhibited a sensitivity of 82.8% and a specificity of 71.4%. The Hosmer-Lemeshow test yielded a non-significant result (*χ*^2^ = 11.13, *P* = 0.194), indicating good calibration of the model. As shown in **Supplementary Table S2**, two baseline GM features remained as independent predictors of moderate-to-severe CRF after adjusting for clinical covariates: a higher abundance of *Veillonella* (OR = 1.860, 95% CI: 1.008–3.434, p=0.047) and a lower relative abundance of the genus *Phascolarctobacterium* (OR = 0.578, 95% CI: 0.343–0.973, p=0.039). Among clinical factors, only pain was independently associated with higher odds of severe fatigue.

#### 3.2.4 GM functional gene analysis

There was no significant difference in grade 1–2 Kyoto Encyclopedia of Genes and Genomes (KEGG) pathways between the Y0 and Y1 groups. A total of 328 class 3 KEGG pathways were analyzed. Nine class 3 KEGG pathways were significantly different (*P <* 0.05) between the Y0 and Y1 groups. These pathways involved amino acid metabolism, nucleotide metabolism, citric acid cycle, and bacterial infection (**Table 2**).

**Table 2:** A significant difference in relative abundance between Y0 and Y1 group in level 3 KEGG pathways.

KEGG pathways	Y0 Mean (SD)	Y1 Mean (SD)	*P*
*Staphylococcus aureus* infection	2.04 × 10^-3^(1.62 × 10^-3^)	3.47 × 10^-3^(3.32 × 10^-3^)	0.019
Bladder cancer	4.35 × 10^-4^(1.18 × 10^-3^)	1.10 × 10^-3^(1.91 × 10^-3^)	0.043
Histidine metabolism	6.19 × 10^-1^(4.84 × 10^-2^)	5.95 × 10^-3^(6.49 × 10^-2^)	0.046
Phosphotransferase system	4.60 × 10^-1^(2.05 × 10^-1^)	5.44 × 10^-1^(2.31 × 10^-1^)	0.049
Folate biosynthesis	3.67 × 10^-1^(4.12 × 10^-2^)	3.84 × 10^-1^(5.36 × 10^-2^)	0.047
Transcription machinery	1.06 × 10^-0^(1.76 × 10^-1^)	1.00 × 10^-0^(1.76 × 10^-1^)	0.043
Glyoxylate and dicarboxylate metabolism	5.47 × 10^-1^(3.53 × 10^-2^)	5.62 × 10^-1^(6.61 × 10^-2^)	0.046
Citrate cycle (TCA cycle)	5.82 × 10^-1^(8.76 × 10^-2^)	5.53 × 10^-1^(1.11 × 10^-1^)	0.048
Melanogenesis	3.79 × 10^-6^(1.50 × 10^-5^)	3.03 × 10^-7^(1.11 × 10^-6^)	0.046

TCA, the tricarboxylic acid; Y1, VAFS≥4 points in the third cycle of chemotherapy; Y0, VAFS<4 points in the third cycle of chemotherapy.

### 3.3 Associations among GM, neuroimmune-endocrine indicators, and CRF

Thirteen patients from the original cohort (n = 100) were stratified into two groups based on changes in CRF during chemotherapy: a CRF-decreased/unchanged group (n = 6) and a CRF-increased group (n = 7). Comparative analysis indicated no significant differences in sociodemographic or clinical characteristics between the pilot cohort (n = 13) and the overall cohort (n = 100) (all *P* > 0.05), verifying the representativeness of the randomly selected sub-sample (**Supplementary Table 3**, **Supplementary Table S3**).

#### 3.3.1 GM and CRF

At the phylum level, the CRF-increased group showed a significant association with a decrease in the abundance of the *Firmicutes* phylum compared with the CRF-decreased/unchanged group (*P* = 0.090). At the genus level, a significant reduction in *Blautia* abundance was observed in the CRF-increased group (*P* = 0.011). No significant differences were observed between the groups at other taxonomic levels or in GM alpha diversity (**Table 3**).

**Table 3:** Relationship of cancer related fatigue with change in the gut microbiota and neuroimmune-endocrine indicators of breast cancer patients during chemotherapy (n=13).

Variable	CRF (mean ± SD)		*p*-value^a^
	increased	decreased/no change	
Gut Microbiota			
Phylum^c^			
Firmicutes	-0.25 ± 0.18	-0.18 ± 0.21	0.049
Genus^d^			
Blautia	-0.01 ± 0.01	0.01 ± 0.02	0.011
Chao1 index	-83.30 ± 54.89	-202.76 ± 130.85	0.555
Shannon index	0.01 ± 1.03	0.09 ± 1.14	0.803
Simpson index	0.01 ± 0.01	0.02 ± 0.02	0.242^b^
Neuroimmune-Endocrine Indicators			
IL-6	-3375.84 ± 1798.84	202.39 ± 173.25	0.517
BDNF	-35.11 ± 13.68	-4.08 ± 2.60	0.035
NGF	-238.02 ± 935.25	357.91 ± 674.53	0.608
OxLDL	881.52 ± 89.67	1125.81 ± 101.03	0.103
GABA	-1.67 ± 0.40	-1.01 ± 0.24	0.174
Cit	-52.90 ± 12.51	-44.86 ± 10.14	0.624
LPS	-3.74 ± 0.90	-3.26 ± 0.58	0.659

a *p*-values were determined via *t*-test unless marked with a.

b , which designates that *p*-value was determined via Mann-Whitney U test.

c relative abundance of phylum-level flora.

d relative abundance of genus-level flora.

#### 3.3.2 Neuroimmune-endocrine indicators and CRF

Compared with the CRF-decreased/unchanged group, the CRF-increased group exhibited a significant reduction in serum brain-derived neurotrophic factor (BDNF) concentration (*P* = 0.035). No significant associations were found with other neuroimmune-endocrine markers. Notably, interleukin-6 (IL-6) and nerve growth factor (NGF) levels declined in the CRF-increased group, but showed an average increase during chemotherapy in the CRF-decreased/unchanged group (**Table 3**).

#### 3.3.3 Gut microbiota, neuroimmune-endocrine indicators, and CRF

**Figure 3:**
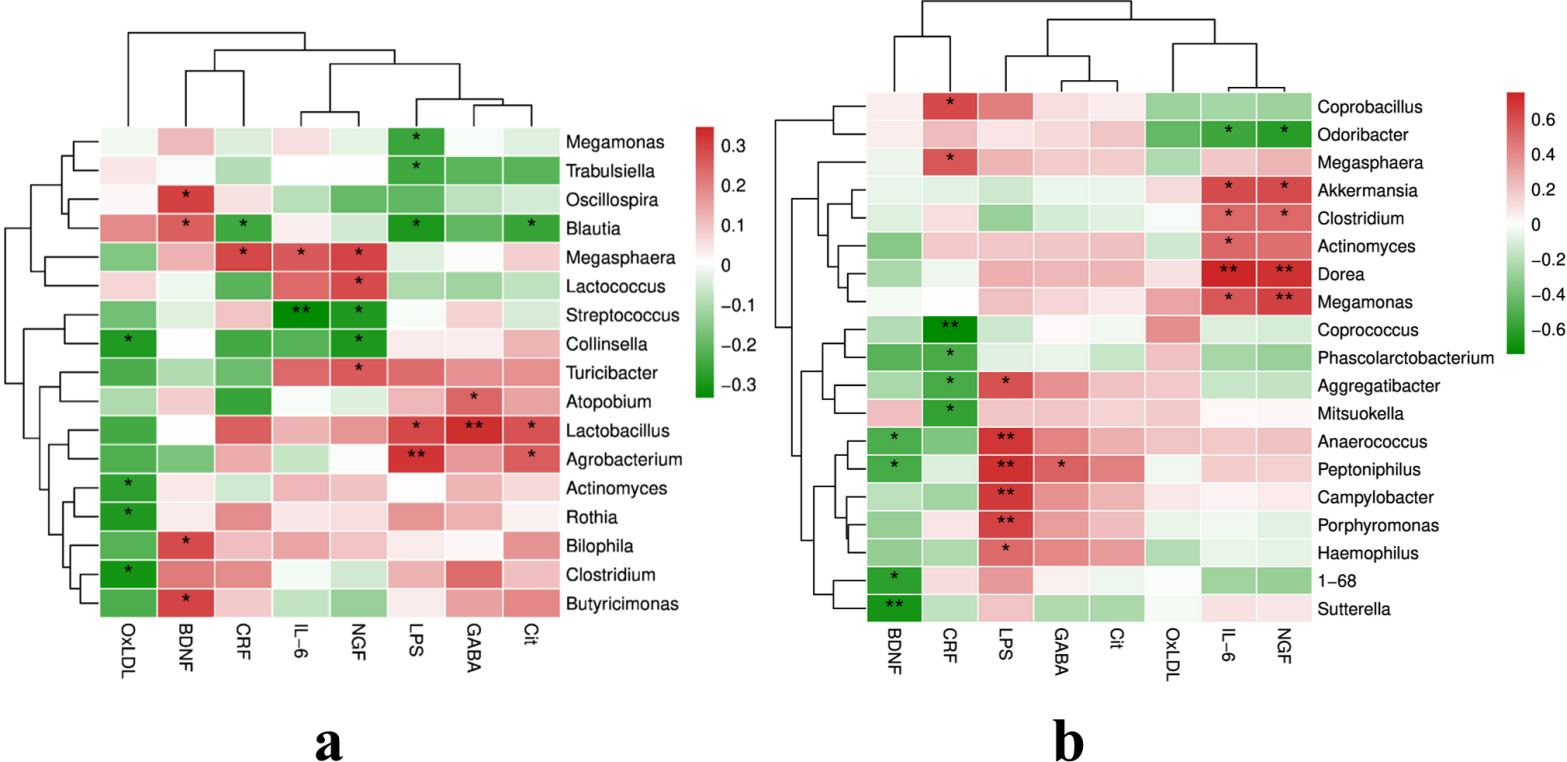
Spearman's correlation analysis between the gut bacteria genus, neuroimmune-endocrine indicators and CRF Note: Heat plot shows the correlation between the relative abundance of bacteria at the genus level, neuroimmune-endocrine indices and CRF in BC patients before chemotherapy **(a)** and during the third cycle of chemotherapy **(b)**. In the heat plot, positive correlations are depicted in red, while negative correlations are represented in green. (*p<0.05, ** p <0.01).

The associations between genus-level relative abundances, CRF scores, and neuroimmune-endocrine indicators are presented in **Figure 3**. Before chemotherapy, the relative abundance of *Blautia* exhibited significant negative correlations with CRF scores, serum LPS concentrations, and serum citrulline (Cit) levels, and a significant positive correlation with serum BDNF concentrations. Conversely, the relative abundance of *Megasphaera* displayed significant positive correlations with CRF scores and IL-6 and NGF serum concentrations. During the third chemotherapy cycle, the relative abundance of *Aggregatibacter* demonstrated a significant negative correlation with CRF scores and a significant positive correlation with serum LPS concentrations.

## 4 Discussion

This study investigated the relationship between pre-chemotherapy GM characteristics and CRF in BC patients during treatment, as well as the dynamic changes among GM, neuroimmune-endocrine indicators, and CRF over the course of chemotherapy. Our results indicate that, compared to BC patients with lower CRF during chemotherapy, those experiencing moderate-to-severe CRF exhibited distinct baseline microbial patterns and functional gene pathways in the gut microbiota. Further analysis revealed that chemotherapy induced gut dysbiosis in BC patients, and a reduction in the abundance of short-chain fatty acid (SCFA)/butyrate-producing bacteria—such as Blautia and the phylum Firmicutes—along with decreased serum BDNF concentration during chemotherapy, was associated with increased CRF severity. These findings support the existence of a potential link among gut microbiota, neuroendocrine markers, and CRF in BC patients undergoing chemotherapy.

Patients who developed moderate-to-severe CRF exhibited distinct baseline gut microbiota characteristics prior to treatment, specifically a significantly increased Bacteroidetes/Firmicutes (B/F) ratio and an elevated relative abundance of potentially pathogenic and pro-inflammatory microorganisms. These baseline microbial signatures suggest an association between gut microbiota profiles and susceptibility to severe CRF, indicating their potential predictive value for fatigue severity at the third chemotherapy cycle, a time point when CRF burden typically peaks. This peak is clinically critical, as the cumulative toxicity of chemotherapy agents often maximizes physical and psychological strain by this stage. Moderate-to-severe fatigue at this juncture can lead to dose reductions or treatment discontinuation, directly impacting therapeutic efficacy and prognosis. Therefore, early identification of high-risk patients based on pre-chemotherapy microbiota profiles holds significant clinical importance for timely intervention. Similar observations have been reported in chronic fatigue syndrome, where dysbiosis characterized by increased pro-inflammatory taxa and reduced short-chain fatty acid (SCFA)-producing bacteria contributes to fatigue severity (23). Our study supported this parallel, emphasizing that chemotherapy-induced microbial disruptions may exacerbate systemic inflammation and metabolic imbalance, thereby promoting fatigue.

Our findings on serum brain-derived neurotrophic factor (BDNF) reductions in patients with worsening CRF are in line with previous studies indicating a relationship between gut microbiota and neurotrophic signaling. Ahmed et al. (24) demonstrated that alterations in gut microbial composition can modulate BDNF expression and influence behavior independent of direct neural transmission. Furthermore, meta-analyses have highlighted the role of SCFA-producing bacteria in upregulating BDNF and promoting neuroplasticity (25), supporting our hypothesis of a gut-brain axis (GBA)-mediated mechanism in CRF. Despite numerical elevations of IL-6, LPS, and OxLDL in patients with aggravated CRF compared to the ameliorated group, statistical significance was not attained. This contrasts with associations between elevated IL-6 and long-term CRF risk in survivors, a discrepancy possibly reflecting distinct inflammatory phases (26). An acute, transient increase in inflammatory markers post-chemotherapy might reflect an effective immune or tissue-stress response, not necessarily correlating with sustained fatigue. Pre-treatment levels of IL-6 along with other inflammatory markers have also been demonstrated to predict the occurrence of CRF of BC cancer during a 2-year follow-up (27). Meanwhile, elevated LPS may be more directly relevant via gut-brain axis mechanisms. Experimental evidence shows LPS suppresses hippocampal BDNF expression (28). Thus, chemotherapy-induced dysbiosis may elevate systemic LPS, contributing to CRF via BDNF suppression—a hypothesis consistent with our observed GM changes and lower serum BDNF in severe fatigue, warranting further validation.

**Figure 4:**
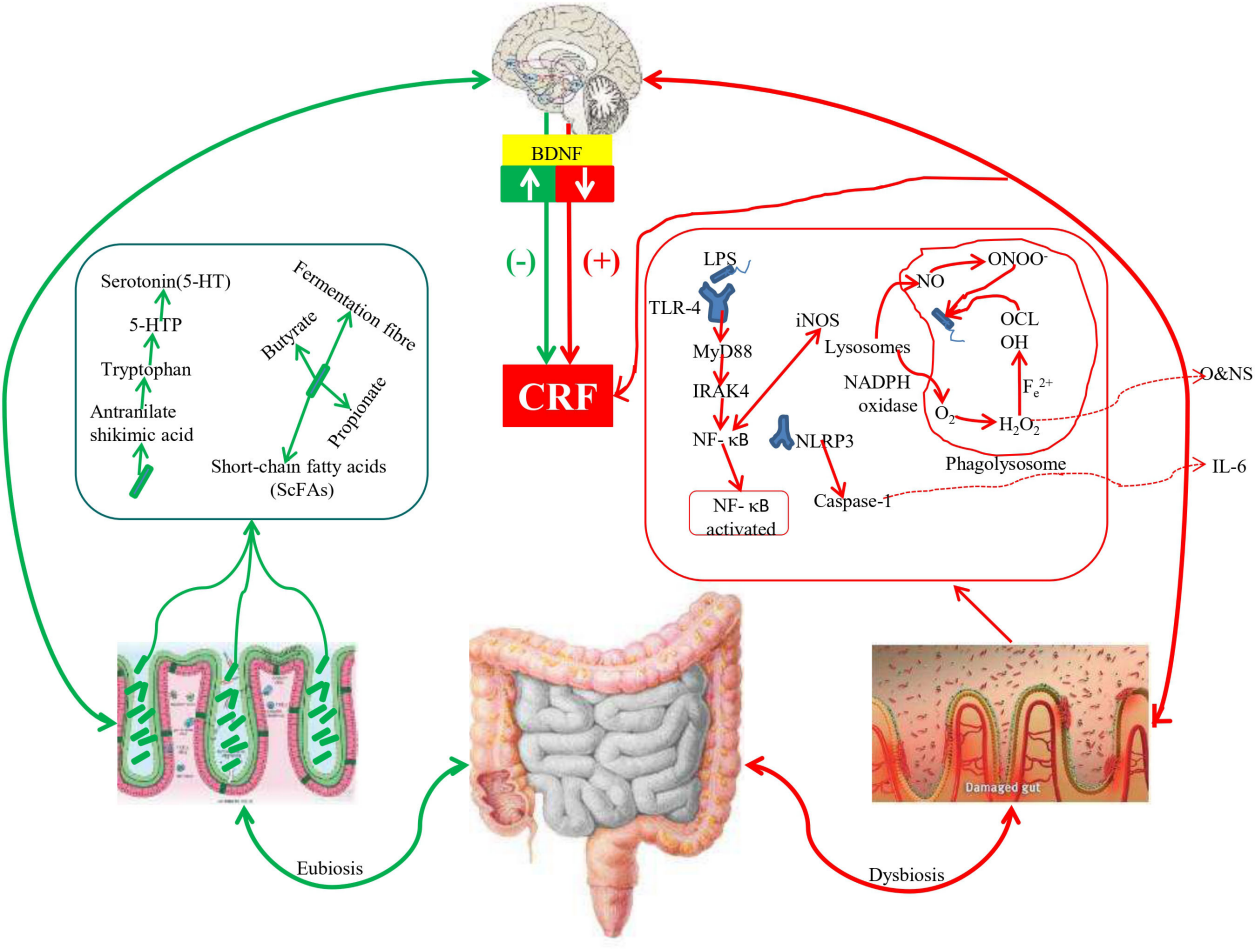
Mechanism of gut microbiota influences CRF via the gut-brain axis during chemotherapy Note: Red upward arrows indicate an increase, while blue downward arrows indicate a decrease. Solid arrows depict direct or established relationships, and dashed arrows represent potential or indirect pathways. BDNF, Brain-Derived Neurotrophic Factor; CRF, Cancer-Related Fatigue; IL-6, Interleukin-6; LPS, Lipopolysaccharide; TLR4, Toll-like Receptor 4.

The findings of this study provide clues for elucidating the mechanisms by which gut microbiota influences CRF via the gut-brain axis during chemotherapy. The underlying mechanisms are illustrated in the **Figure 4**. First, gut dysbiosis directly weakens protective pathways. Reduced production of SCFAs, particularly butyrate, diminishes not only their ability to cross the blood-brain barrier and directly upregulate BDNF but also compromises their role in maintaining intestinal barrier integrity. Second, the dysbiotic microbiota may activate inflammatory pathways. An increase in Gram-negative bacteria, including *Proteobacteria*, could elevate the load of LPS. As depicted in the figure, LPS can trigger a systemic inflammatory response, leading to the release of pro-inflammatory cytokines such as IL-6. These inflammatory mediators can directly induce sickness behavior and fatigue and may indirectly suppress BDNF expression, creating a vicious cycle of inflammation and insufficient neurotrophic support. Finally, these pathways converge to contribute to CRF. The decrease in serum BDNF levels observed in our study likely represents a key outcome resulting from the combined effects of reduced SCFAs and enhanced systemic inflammation. BDNF, as a central regulator of neuronal energy metabolism and plasticity, may directly contribute to the onset of central fatigue when its levels are diminished.

Overall, our study confirms and extends prior observations, providing additional evidence that baseline GM characteristics may serve as predictive biomarkers for CRF and highlighting the potential of microbiota-targeted strategies to mitigate chemotherapy-related fatigue.

### 4.1 Implications for practice

In terms of clinical translation, our study suggests that baseline GM profiling could be integrated into risk stratification protocols to identify patients at high risk for severe CRF before chemotherapy begins. Such patients could be prioritized for multimodal supportive care, including dietary counseling (e.g., high-fiber, prebiotic-rich diets), probiotic supplementation, and closer monitoring of fatigue and inflammatory markers. Additionally, serum BDNF may serve as an accessible biomarker for tracking CRF progression and response to microbiota-targeted interventions.

### 4.2 Limitations

This study has several limitations. First, regarding study design and sample, this was a single-center investigation, and the cohort was recruited from one city. Caution is warranted when generalizing the findings to other populations, as geographical, dietary, and population-specific characteristics may influence baseline gut microbiota profiles. Second, concerning measurement and confounding factors, although we measured key gut-brain axis mediators and controlled for major clinical variables, residual confounding from factors that are difficult to quantify precisely cannot be entirely ruled out. For instance, detailed individual dietary patterns, micronutrient status (e.g., minerals) may influence the levels of neuroimmune-endocrine markers and CRF, potentially attenuating the strength of the observed associations. Third, pertaining to mechanistic exploration and causal inference, while changes in peripheral blood neurotransmitter levels and BDNF secretion were not comprehensively analyzed over time, which may provide further insight into the mechanisms underlying CRF development. Future research employing multi-center longitudinal designs with larger samples, repeated standardized sampling, and detailed nutritional assessments is needed to validate the causal role of gut microbiota alterations in CRF progression and to elucidate the underlying dynamic mechanisms.

## 5 Conclusions

This prospective study demonstrates that baseline gut microbiota (GM) profiles are potential predictors of cancer-related fatigue (CRF) severity in patients with breast cancer undergoing chemotherapy. An elevated Bacteroidetes/Firmicutes ratio and higher abundances of Proteobacteria and Enterobacteriaceae were associated with an increased risk of moderate-to-severe CRF. Chemotherapy-induced depletion of Firmicutes and Blautia may exacerbate CRF by impairing short-chain fatty acid production, compromising intestinal barrier function, and promoting systemic inflammation. Additionally, reduced brain-derived neurotrophic factor (BDNF) levels suggest a possible gut-brain axis–mediated mechanism. These findings provide a rationale for developing GM-targeted interventions to mitigate chemotherapy-related fatigue and highlight the need for validation in larger, multicenter studies.

## Data Availability

The original contributions presented in the study are included in the article/**Supplementary Material**. Further inquiries can be directed to the corresponding authors.
