# Supplementary material for: Chemotherapy-induced gut microbiota dysbiosis exacerbates cancer-related fatigue in breast cancer patients via neuroimmune-endocrine indicators
Source: Front Oncol. 2026 Jan 26;16:1710457. doi: 10.3389/fonc.2026.1710457 (PMC12883358; doi:10.3389/fonc.2026.1710457)
Supplement: Supplementary file 1 [file DataSheet1.docx]

**Supplementary Material**

***1. Supplementary Table***

**Table S1.** Comparison of CRF between two groups of breast cancer patients

| **CRF** | **Time** | **Group (mean ± SD)** | | ***t*** | ***p*** |
| --- | --- | --- | --- | --- | --- |
|  |  | **Y0** | **Y1** |  |  |
| Physical fatigue | T0 | 14.76±5.08 | 15.93±5.25 | -1.114 | 0.268 |
|  | T1 | 15.62±3.54 | 20.48±3.10 | -7.291 | 0.000 |
| Emotional fatigue | T0 | 5.17±2.81 | 6.59±2.99 | -2.402 | 0.018 |
|  | T1 | 6.29±2.86 | 7.31±1.98 | -2.117 | 0.037 |
| Cognitive fatigue | T0 | 3.57±3.35 | 3.88±3.61 | -0.434 | 0.665 |
|  | T1 | 4.10±2.73 | 5.17±3.63 | -1.618 | 0.109 |
| Fatigue total score | T0 | 23.50±9.21 | 26.40±9.35 | -1.539 | 0.127 |
|  | T1 | 26.00±6.32 | 32.97±6.42 | -5.391 | 0.000 |
| Degree of fatigue | T0 | 2.50±2.41 | 2.81±2.20 | -0.669 | 0.505 |
|  | T1 | 2.36±0.79 | 5.60±1.08 | -16.580 | 0.000 |

Note: T0, before chemotherapy; T1, the third cycle of chemotherapy; Y0, VAFS<4 points in the third cycle of chemotherapy; Y1, VAFS≥4 points in the third cycle of chemotherapy.

**Table S2.** Multivariable logistic regression analysis of factors predicting moderate-to-severe CRF.

| **Predictor** | ***β*** | **OR** | **95% CI for OR** | ***P*** |
| --- | --- | --- | --- | --- |
| Age | 0.032 | 1.033 | 0.963-1.108 | 0.365 |
| BMI | -0.103 | 0.902 | 0.777-1.048 | 0.177 |
| Menstrual condition | -0.064 | 0.938 | 0.421-2.094 | 0.876 |
| Family history | -0.127 | 0.880 | 0.096-8.085 | 0.910 |
| Cancer metastasis | -0.248 | 0.781 | 0.227-2.688 | 0.695 |
| Cancer Staging | -0.116 | 0.890 | 0.247-3.212 | 0.859 |
| Combined radiotherapy | 0.327 | 1.386 | 0.264-7.287 | 0.700 |
| Other diseases | -0.198 | 0.820 | 0.618-1.089 | 0.171 |
| Pain score | 1.453 | 4.277 | 1.512-12.097 | 0.006 |
| Sleep quality | -0.313 | 0.731 | 0.414-1.289 | 0.279 |
| *Gammaproteobacteria* | 0.598 | 1.819 | 0.935-3.537 | 0.078 |
| *Veillonella* | 0.621 | 1.860 | 1.008-3.434 | 0.047 |
| *Ralstonia* | -4.436 | 0.012 | 0.000-3.284 | 0.122 |
| *Phascolarctobacterium* | -0.548 | 0.578 | 0.343-0.973 | 0.039 |

Note: OR, Odds Ratio; CI, confidence interval.

**Table S3.** Comparison of sociodemographic and clinical information between two cohorts of breast cancer patients

| **Variable [*n* (%)]** | **P0 (*n*=100)** | **P1 (*n*=13)** | ***t/f*** | ***P*** |
| --- | --- | --- | --- | --- |
| Age, mean ± SD, yr | 50.95±11.62 | 55.62±9.67 | -1.385 ^a^ | 0.169 |
| Religious belief |  |  | 0.741 ^b^ | 0.406 |
| Yes | 6 (6.0) | 1 (7.7) |  |  |
| No | 94 (94.0) | 12 (92.3) |  |  |
| Marital status |  |  | 0.000 ^b^ | 1.000 |
| Married/Cohabitation | 96（96.0） | 13（100.0） |  |  |
| Single/Divorced/Widowed | 4（4.0） | 0 |  |  |
| Educational level |  |  | 1.103 ^b^ | 0.914 |
| Elementary school and below | 33（33.0） | 5（38.5） |  |  |
| Middle school | 53（53.0） | 7（53.8） |  |  |
| Junior college and above | 14（14.0） | 1（7.7） |  |  |
| Employment status |  |  | 1.482 ^b^ | 0.456 |
| Employed | 33（33.0） | 3（23.1） |  |  |
| Retired | 26（26.0） | 7（53.8） |  |  |
| Unemployed | 41（41.0） | 1（7.7） |  |  |
| Place of residence |  |  | 1.011 ^b^ | 0.929 |
| Urban | 74（74.0） | 10（76.9） |  |  |
| Rural | 26（26.0） | 3（23.7） |  |  |
| Lifestyle |  |  | 0.000 ^b^ | 1.000 |
| Living alone | 5（5.0） | 0 |  |  |
| Living with family | 95（95.0） | 13（100.0） |  |  |
| Per capita monthly household income | |  | 0.935 ^b^ | 0.789 |
| ＜3000 | 25（25.0） | 3（23.1） |  |  |
| 3000~6000 | 33（33.0） | 4（30.8） |  |  |
| ＞6000 | 42（42.0） | 6（46.2） |  |  |
| BMI, mean ± SD, kg/m^2^ | 23.71±3.76 | 24.01±4.41 | -0.265 ^a^ | 0.792 |
| Menstrual condition |  |  | 0.812 ^b^ | 0.546 |
| Regular menstrual cycle | 40（40.0） | 4（30.8） |  |  |
| Irregular menstrual cycle | 11（11.0） | 0 |  |  |
| Menopause/ Hysterectomy | 49（49.0） | 9（69.2） |  |  |
| Family history |  |  | 0.000 ^b^ | 1.000 |
| Yes | 6（6.0） | 0 |  |  |
| No | 94（94.0） | 13（100.0) |  |  |
| Cancer metastasis |  |  | 0.857 ^b^ | 0.635 |
| Yes | 32（32.0） | 5（38.5） |  |  |
| No | 68（68.0） | 8（61.5） |  |  |
| Cancer Staging |  |  | 2.364 ^b^ | 0.098 |
| Ⅰ | 9（9.0） | 1（7.7） |  |  |
| Ⅱ | 82（82.0） | 12（92.3） |  |  |
| Ⅲ | 9（9.0） | 0 |  |  |
| Combined radiotherapy |  |  | 0.000 ^b^ | 1.000 |
| Yes | 10（10.0） | 0 |  |  |
| No | 90（90.0） | 13（100.0） |  |  |
| Other diseases |  |  | 2.426 ^b^ | 0.089 |
| Hypertension | 24（24.0） | 2（15.4） |  |  |
| Diabetes | 2（2.0） | 0 |  |  |
| None | 74（74.0） | 11（84.6） |  |  |
| Pain score |  |  | 1.221 ^b^ | 0.739 |
| Painless | 54（54.0） | 8（61.5） |  |  |
| Mild pain | 43（43.0） | 5（38.5） |  |  |
| Moderate pain | 3（3.0） | 0 |  |  |
| Sleep quality |  |  | 1.230 ^b^ | 0.727 |
| Good | 25（25.0） | 2（15.4） |  |  |
| Average | 67（67.0） | 10（76.9） |  |  |
| Poor | 8（8.0） | 1（7.7） |  |  |

Note: P0, 100 BC patients in prospective study; P1, 13 BC patients in pilot study; TC, docetaxel and cyclophosphamide; EC, paclitaxel and carboplatin; AC, doxorubicin plus cyclophosphamide; a, t-test; b, Fisher's exact test.

***2. Supplementary Figure***


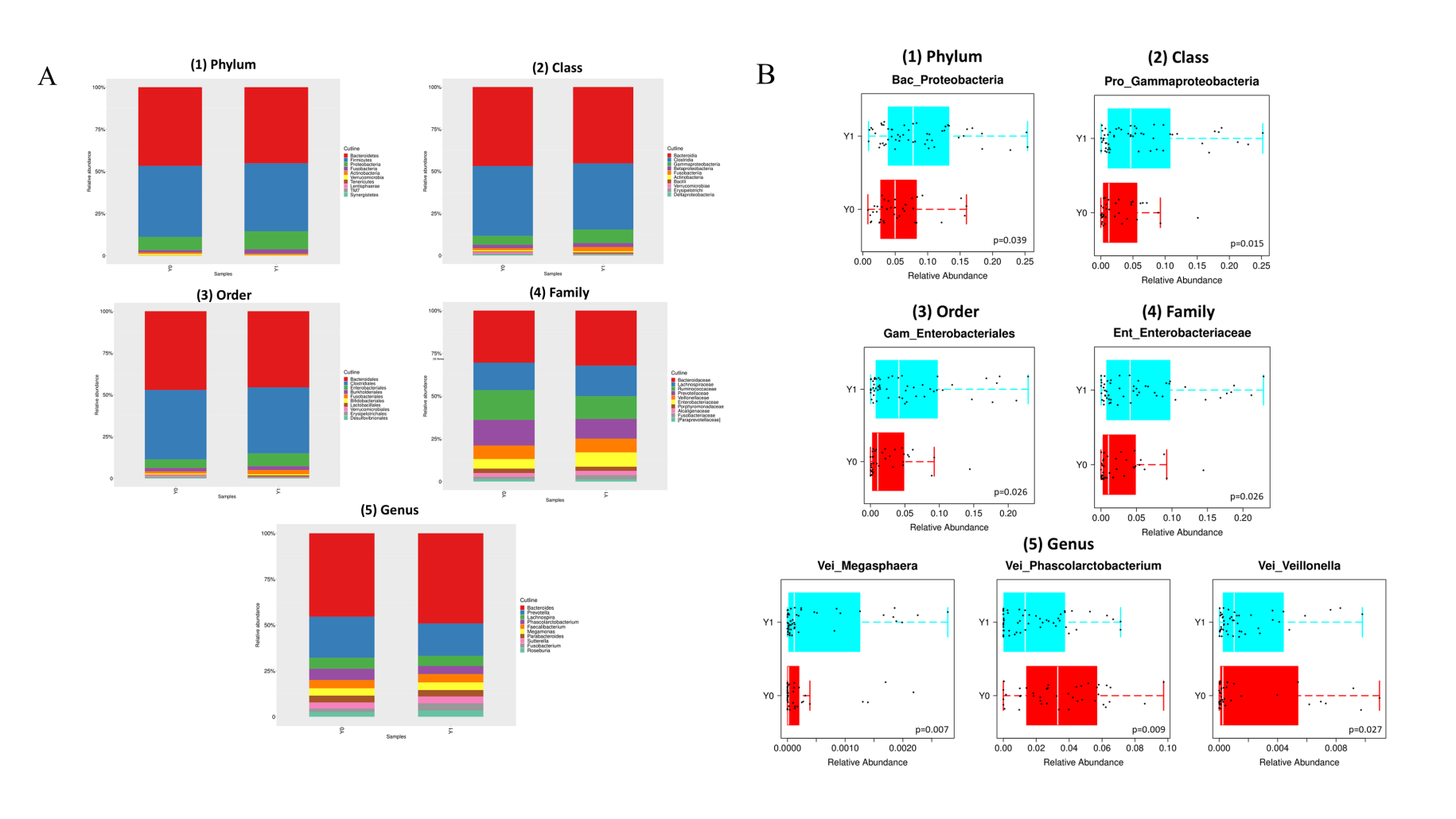
**Figure S1.** Composition of the gut microbiota and the analysis of species differences at the phylum, class, order, family and genus levels between the two groups.

Note: (A1, B1) Bar charts and the analysis of species differences at the phylum level; (A2, B2) Bar charts and the analysis of species differences at the class level; (A3, B3) Bar charts and the analysis of species differences at the order level; (A4, B4) Bar charts and the analysis of species differences at the family level; (A5, B5) Bar charts and the analysis of species differences at the genus level. Y0, VAFS<4 points in the third cycle of chemotherapy; Y1, VAFS≥4 points in the third cycle of chemotherapy.


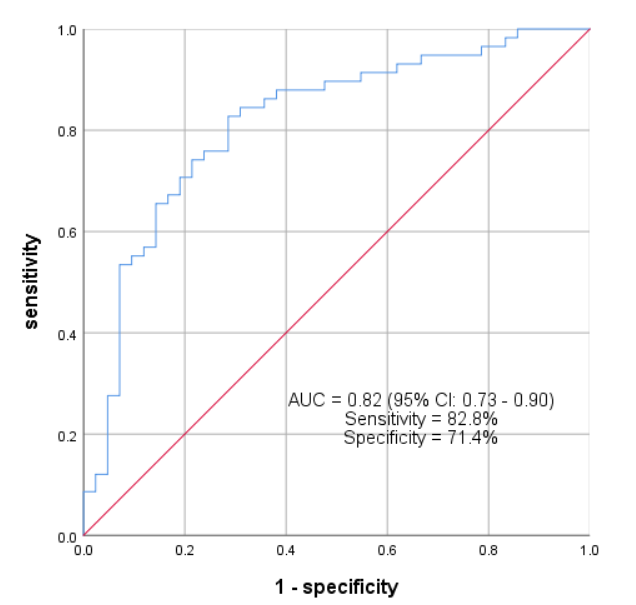


**Figure S2.** ROC curve of the logistic regression model for predicting moderate-to-severe cancer-related fatigue.

Note: ROC, receiver operating characteristic; AUC, area under the curve.
